# Exploring resistance to initial chemotherapy in small cell lung cancer: The role of bone metastasis and other clinicopathologic characteristics

**DOI:** 10.1097/MD.0000000000041953

**Published:** 2025-03-21

**Authors:** Yulong He, Xiaorong Tang, Fang Yang, Qinling Jiang, Lin Deng, Wenwang Lang

**Affiliations:** a Department of Oncology, Nanxishan Hospital of the Guangxi Zhuang Autonomous Region, Guilin, Guangxi, China; b Department of Spine Surgery, Guilin People’s Hospital, Guilin, Guangxi, China; c Department of Pharmacy, Nanxishan Hospital of the Guangxi Zhuang Autonomous Region, Guilin, Guangxi, China.

**Keywords:** chemosensitivity, drug resistance, molecular subtypes, osteoimmuno-oncology, small cell lung cancer

## Abstract

Clinically, approximately 10% to 20% of small cell lung cancer (SCLC) patients do not respond well to initial platinum-based first-line chemotherapy. Knowledge about the clinicopathologic characteristics of these primary drug-resistant populations is limited. This study aimed to explore the clinicopathologic characteristics in SCLC populations insensitive to initial chemotherapy. This study enrolled SCLC patients with insensitivity to initial chemotherapy and analyzed their clinicopathological characteristics. Binary logistic regression analysis was used to determine the independent factors that influence chemosensitivity. The study evaluated 142 cases to determine the clinicopathologic characteristics of SCLC populations with insensitivity to initial chemotherapy. Between the chemotherapy-insensitive group (n = 32) and the chemotherapy-sensitive group (n = 110), no significant differences were observed in sex, age, smoking status, tumor size, lymph-node metastasis, vascular invasion, carcinomatous lymphangitis, mediastinal invasion, superior vena cava syndrome, tumor stage, brain metastases, pleural metastasis, lung metastasis, adrenal metastasis, or the immunohistochemical markers cytokeratin, synaptophysin, chromogranin A, thyroid transcription factor-1, and Ki-67 (all *P* > .05). However, significant differences in liver metastasis (*P* = .005), bone metastasis (*P* < .001), and neural cell adhesion molecule expression (*P* = .027) were identified. Binary logistic regression analysis revealed that bone metastasis (*P* = .008) was an independent high-risk factor for insensitivity to initial first-line chemotherapy. Bone metastasis is an independent high-risk factor for insensitivity to initial chemotherapy in SCLC. Enhancing our understanding of SCLC biology and osteoimmuno-oncology could identify new vulnerabilities and better define patient populations that may benefit from tailored clinical treatments to overcome drug resistance.

## 1. Introduction

Lung cancer remains the deadliest malignancy worldwide, accounting for more than a quarter of all cancer deaths annually.^[1]^ Small cell lung cancer (SCLC), approximately 13% to 15% of all primary lung neoplasms,^[2]^ characterized by its aggressive neuroendocrine nature with fast proliferation rate, strong invasiveness, intrinsic drug resistance, and early dissemination, which lead to a high relapse rate, limited therapeutic options, and poor prognosis.^[3,4]^

In the personalized and precision therapy era, SCLC presents substantial challenges, partly due to its inter- and intra-tumoral heterogeneity. This heterogeneity leads to unique clinicopathologic features that are not yet fully understood. Historically, SCLC clinical trials have often involved unselected populations, resulting in predictably disappointing outcomes.^[5]^ Despite ongoing efforts to innovate, advances in SCLC systemic therapy have stagnated for over 3 decades.^[6]^ The standard treatment options for SCLC currently used as first-line therapy include platinum-based chemotherapy, such as cisplatin plus etoposide oroplatin plus etoposide, and the treatment strategy has not changed significantly over the years.^[7]^ Chemotherapy, while initially effective, quickly leads to drug resistance and disease relapse. Median overall survival for extensive-stage SCLC is approximately 10 to 12 months, compared to approximately 17 months for limited-stage disease.^[8]^ Recent approvals of immune checkpoint inhibitors for extensive or recurrent SCLC mark a modest advance, offering some patients an approximate 3-month prolongation of survival when combined with first-line chemotherapy.^[9,10]^ However, only a minor subset of patients, particularly those with a specific tumor microenvironment, show significant responses to immunotherapy, and resistance to immune checkpoint inhibitors treatment alone may develop.^[11,12]^

About 10% to 20% of SCLC patients initially exhibit poor responses to first-line chemotherapy, indicating primary resistance.^[13]^ Unfortunately, our understanding of the clinicopathological characteristics of these primarily resistant populations is limited. Although research on drug resistance mechanisms continues, identifying specific, predictable, and significant mechanisms remains elusive.

Considering SCLC’s persistent resistance to chemotherapy and immunotherapy, this study aimed to investigate the clinicopathologic features and potential strategies for overcoming primary resistance in these populations, suggesting new approaches for future treatment strategies.

## 2. Materials and methods

### 2.1. Patients and outcome evaluation

This retrospective study identified patients treated for SCLC at the Nanxishan Hospital of the Guangxi Zhuang Autonomous Region between April 2015 and January 2024. The inclusion criteria were as follows: (1) SCLC diagnosis according to the 2015 World Health Organization (WHO) Classification of Lung Tumors,^[14]^ (2) completion of at least 2 consecutive cycles of a platinum-based 2-drug regimen (platinum plus etoposide) as first-line chemotherapy, and (3) undergoing chest imaging and tissue immunohistochemical testing. Exclusion criteria: (1) presence of concomitant primary tumors, (2) presence of acute infectious diseases, and (3) loss of follow-up.

CT scans were performed after 2 chemotherapy cycles to assess short-term effectiveness, and responses were evaluated using the Response Evaluation Criteria in Solid Tumors (RECIST) version 1.1.^[15]^ Treatment responses were categorized as complete response (CR): all target lesions disappear; partial response (PR):the sum of the longest diameters of the baseline lesions is reduced by ≥ 30%; progressive disease (PD): the sum of the longest diameters of the baseline lesions increases by ≥ 20%, including an absolute increase of 5 mm or the appearance of new lesions; and stable disease (SD): the sum of the longest diameters of the baseline lesions is reduced but does not reach the criteria for PR, or it increases but does not reach the criteria for PD. CR and PR were indicators of chemosensitivity and classified patients into a chemotherapy-sensitive group, SD and PD were resistance indicators, placing patients in a chemotherapy-insensitive group.^[13]^

The clinical tumor stage was determined using the Eighth Edition TNM Stage Classification for Lung Cancer by the American Joint Commission on Cancer (AJCC).^[16]^ The follow-up ended on January 31, 2024.

### 2.2. Data collection

Demographic and clinical data were extracted from medical records, including sex, age, smoking history, tumor stage, tumor diameter, presence of lymphatic invasion, vascular invasion, carcinomatous lymphangitis, mediastinal invasion, superior vena cava compression (SVCC) syndrome, distant metastasis, and immunohistochemical indices. Key immunohistochemical markers analyzed included cytokeratin (CK), synaptophysin (Syn), neural cell adhesion molecule (CD56), thyroid transcription factor-1 (TTF-1), chromogranin A (CgA), and Ki-67.^[17]^

### 2.3. Immunohistochemistry

Immunohistochemical EnVision method was used to detect Syn, CD56, TTF-1, and Ki-67. Cancer tissues were cut into 3-micrometer-thick sections. The Testing process is carried out in strict accordance with the instructions.CK mouse antihuman monoclonal antibody, Syn mouse antihuman monoclonal antibody, CD56 mouse antihuman monoclonal antibody, TTF-1 mouse antihuman monoclonal antibody, Ki-67 mouse antihuman monoclonal antibody were purchased from Fuzhou New Step Biotechnology Development Co., Ltd. Immunohistochemical staining was reviewed by 2 experienced pathologists. Discordant cases were assessed by a third physician and eventually discussed to reach a consensus. Syn, CD56, TTF-1, KI-67 positive judgment criterion.^[18]^

### 2.4. Statistical analysis

Differences in clinical characteristics and immunophenotypes between chemotherapy-sensitive and -insensitive groups were assessed. Continuous variables are expressed as mean and standard deviation, and were analyzed using the *t* test or the Wilcoxon Mann–Whitney test. Categories are expressed as numbers and percentages, and they were evaluated using the Pearson chi-square test or Fisher exact test. Factors influencing chemosensitivity were identified by binary logistic regression analysis (α = 0.05). All statistical analyses were conducted using the SPSS software package (version 23.0), with *P* < .05 deemed statistically significant.

## 3. Results

### 3.1. Demographic and clinical characteristics

A total of 142 SCLC patients were analyzed, including 32 in the chemotherapy-insensitive group (SD 23/32, 71.9%; PD 9/32, 28.1%) and 110 in the chemotherapy-sensitive group (CR 1/110, 0.9%; PR 109/110, 99.1%). There were no significant differences between the 2 groups in terms of sex (males: 94 in the chemotherapy-sensitive group vs 29 in the chemotherapy-insensitive group; females: 16 in the chemotherapy-sensitive group vs 3 in the chemotherapy-insensitive group; *P* = .450), age (62.04 ± 7.82 years in the chemotherapy-sensitive group vs 60.63 ± 8.15 years in the chemotherapy-insensitive group; *P* = .375), smoking history (smokers: 88 in the chemotherapy-sensitive group vs 27 in the chemotherapy-insensitive group; *P* = .579), and tumor size (≥5 cm: 75 in the chemotherapy-sensitive group vs 19 in the chemotherapy-insensitive group; *P* = .354).

**Figure 1.**
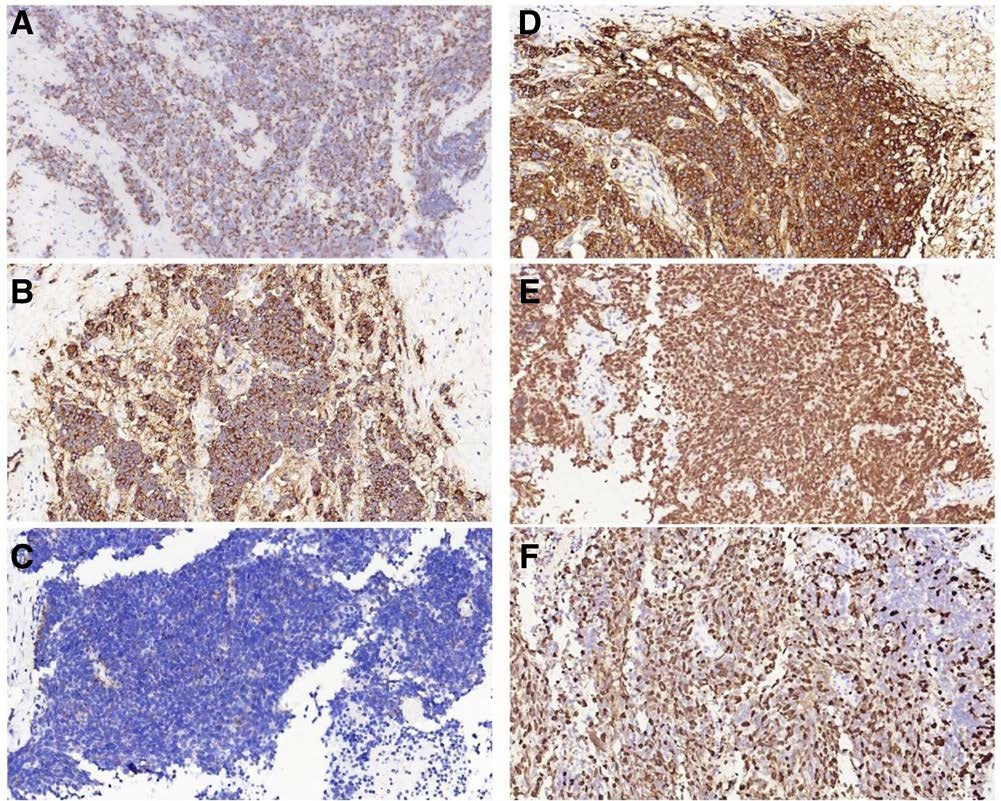
Immunohistochemical staining (×400). (A) Cytokeratin-positive expression in SCLC. (B) CD56-positive expression in SCLC. (C) Chromogranin A-positive expression in SCLC. (D) Syn-positive expression in SCLC. (E) Thyroid transcription factor-1-positive expression in SCLC. (F) Ki-67-positive expression in SCLC. SCLC = small cell lung carcinoma.

In the chemotherapy-insensitive group, 90.6% (29/32) of the patients developed lymph node metastasis, 50% (16/32) with vascular invasion, 21.9% (7/32) had carcinomatous lymphangitis, 12.5% (4/32) had mediastinal invasion, and 9.4% (3/32) had SVCC syndrome. Distant metastases were commonly observed in the liver (34.4%, 11/32), bone (28.1%, 9/32), pleura (21.9%, 7/32), brain (9.4%, 3/32), lung (9.4%, 3/32), and adrenal gland (6.3%, 2/32). There were no significant differences in lymph-node metastasis, vascular invasion, carcinomatous lymphangitis, mediastinal invasion, superior vena cava syndrome, or tumor stage. Details are shown in Table 1 and Figure 1.

**Table 1:** Clinicopathologic features of SCLC between the chemotherapy-insensitive group and the chemotherapy-sensitive group.

	Chemotherapy-sensitive group (n = 110)	Chemotherapy-insensitive group (n = 32)	*x^2^*/t	*P*
Sex				
Male	94	29	0.572	.450
Female	16	3		
Age (years)				
	37–79 (62.04 ± 7.82)	38–74 (60.63 ± 8.15)	-0.890	.375
≥ 60	74	21	0.030	.862
< 60	36	11		
Smoking history				
Yes	88	27	0.308	.579
No	22	5		
Tumor size (cm)				
≥ 5	75	19	0.859	.354
< 5	35	13		
Lymph-node metastasis				
Yes	98	29	0.062	.804
No	12	3		
Vascular invasion				
Yes	49	16	0.297	.586
No	61	16		
Carcinomatous lymphangitis				
Yes	20	7	0.220	.639
No	90	25		
Mediastinal invasion				
Yes	29	4	2.671	.102
No	81	28		
Superior vein cave syndrome				
Yes	12	3	0.062	.804
No	98	29		
Stage				
IV	49	20	3.199	.072
IB-IIIC	61	12		
Liver metastasis				
Yes	14	11	8.008	***.005***
No	96	21		
Bone metastasis				
Yes	6	9	13.485	***.000***
No	104	23		
Brain metastases				
Yes	10	3	0.002	.961
No	100	29		
Pleural metastasis				
Yes	15	7	1.285	.257
No	95	25		
Lung metastasis				
Yes	14	3	0.264	.607
No	96	29		
Adrenal metastasis				
Yes	8	2	0.040	.842
No	102	30		
CK				
Positive	92	31	4.402	.111
Negative	13	0		
None	5	1		
Syn				
Positive	99	27	0.904	.636
Negative	8	4		
None	3	1		
CgA				
Positive	37	4	5.733	.057
Negative	25	8		
None	48	20		
CD56				
Positive	97	29	7.237	***.027***
Negative	2	3		
None	11	0		
TTF-1				
Positive	96	24	3.782	.151
Negative	8	6		
None	6	2		
Ki-67 (%)				
> 50	91	27	1.957	.376
≤ 50	14	2		
None	5	3		

Age was a continuous variable and analyzed using the *t* test. The remaining variables were categorical variables, and evaluated using the Pearson chi-square test. Bold values indicate significant group differences.

CD56 = neural cell adhesion molecule, CgA = chromogranin A, CK = cytokeratin, SCLC = small cell lung cancer, Syn = synaptophysin, TTF-1 = thyroid transcription factor-1. None = no data.

### 3.2. Metastatic and immunohistochemical features

There were significant differences in the incidence of liver metastases (14 in the chemo-sensitive group vs 11 in the chemotherapy-insensitive group; *P* = .005) and bone metastases (6 in the chemotherapy-sensitive vs 9 in the chemotherapy-insensitive group; *P* < .001). In the chemotherapy-insensitive group, the percentages of patients positive for CK, Syn, CD56, TTF-1, and CgA were 100%, 87.1%, 90.6%, 80%, and 33.3%, respectively. Additionally, 93.1% (27/29) of the patients had a high Ki-67 index (>50%). The prevalence of CD56 expression also varied significantly (97 positives in the chemotherapy-sensitive group vs 29 positives in the chemotherapy-insensitive group; *P* = .027). However, no significant differences were observed in brain, pleural, lung, or adrenal metastases, nor the expression of immunohistochemical markers such as CK, Syn, CgA, TTF-1, and Ki-67. Details are shown in Table 1.

### 3.3. Binary logistic regression analysis

Binary logistic regression analysis showed that bone metastasis was a significant independent high-risk factor for chemotherapy insensitivity (exponentiation of the B coefficient [Exp (B)] = 0.125, *P* = .008). This suggests a strong predictive value of bone metastasis in determining chemoresistance in SCLC patients. Although different between groups, liver metastasis, and CD56 expression did not achieve statistical significance in the regression model affecting chemosensitivity. Details are shown in Table 2.

**Table 2:** Binary logistic regression analysis for influencing factors of chemosensitivity.

Risk factor	*B*	SE	Wals	Exp (B)	95% CI	*P*
Liver metastasis						
Yes *vs* no	-0.389	0.632	0.379	0.677	0.196–2.339	.538
Bone metastasis						
Yes *vs* no	-2.075	0.779	7.097	0.125	0.027–0.578	**.008**
CD56						
Positive *vs* negative	–	–	4.410	–	–	.110

Bold value indicates significant group differences.

B = regression coefficient, CI = confidence interval, Exp (B) = exponentiation of the B coefficient, SE = standard error, Wals = Wald χ^2^.

## 4. Discussion

### 4.1. Necessity of the study and characteristics of the included population

This is the first study to evaluate the clinicopathologic characteristics in the SCLC population, specifically unresponsive to platinum-based chemotherapy. SCLC represents a highly heterogeneous group of neuroendocrine tumors, each exhibiting diverse clinical manifestations, pathological features, and prognoses. A detailed investigation of the clinicopathologic characteristics of SCLC populations with initial chemotherapy insensitivity, the underlying mechanisms of primary or acquired drug resistance, and potential therapeutic strategies could significantly advance treatment and clinical outcomes.

Among the 142 cases studied, 32 were chemotherapy-insensitive, representing 22.5% of the sample, which is consistent with a previous study.^[13]^ These chemotherapy-insensitive cases predominantly involved male and older patients, with a mean age of 60 years. In particular, 84.4% of these patients had a history of smoking, suggesting a potential risk factor for SCLC. Most of these cases were diagnosed in advanced TNM stages, reflecting the high malignancy and propensity for distant metastasis in SCLC, frequently affecting the liver (34.4%), pleura (21.9%), bones (28.1%), brain (9.4%), lung (9.4%), and adrenal glands (6.3%), findings consistent with previous research.^[19]^

Immunohistochemical analysis revealed high positivity rates for CK, Syn, CD56, and TTF-1 in SCLC, corroborating previous reports.^[20,21]^ The Ki-67 index, indicative of cell proliferation capacity, has been widely recognized for differentiating SCLC from carcinoids, although it does not distinguish between typical and atypical carcinoids.^[22,23]^

### 4.2. Comparison of clinicopathologic characteristics between chemotherapy-insensitive and -sensitive groups

This retrospective study also compared clinicopathologic characteristics between chemotherapy-insensitive and chemotherapy-sensitive groups. No significant differences were found in terms of sex, age, smoking history, tumor size, lymph node metastasis, vascular invasion, carcinomatous lymphangitis, mediastinal invasion, SVCC syndrome, tumor stage, or metastases (brain, pleura, lung, adrenal) or markers (CK, Syn, CgA, TTF-1, Ki-67). However, Zhu et al^[24]^ reported that while sex and age did not significantly affect short-term efficacy, and chemosensitivity in SCLC was closely related to smoking status and tumor stage. Wang et al^[13]^ observed that the objective response rate was higher in TTF-1-positive patients, suggesting that TTF-1 may be a predictive marker of response to first-line chemotherapy, a finding echoed by another study.^[25]^ This discrepancy was observed primarily in limited-stage SCLC, with no significant correlation between TTF-1 expression and objective response rate in extensive-stage SCLC.^[13]^

Our study also found that significant disparities were observed in liver metastasis, bone metastasis, and CD56 expression. Tumors in different locations may possess distinct biological characteristics, which means that their sensitivities to chemotherapy may vary. Liver and bone metastasis may have different sensitivities to chemotherapy due to different microenvironments and biological characteristics, resulting in the ineffectiveness of chemotherapy.^[26]^ Study found that in most patients, chemotherapy cannot completely eliminate different subsets of circulating tumor cells, including the CD56-positive circulating tumor cell subset, because some of these patients are sensitive to chemotherapy while others seem to be resistant to it. This indicates that there is a certain relationship between CD56 expression and the chemotherapy sensitivity of small cell lung cancer (SCLC), which is worthy of further exploration.^[27]^

Variations in these results may arise from different immunohistochemical techniques and criteria used in other studies, representing a potential confounder. The discrepancies between patient groups and the retrospective, small-scale nature of most studies, including ours, may further contribute to these differences.

### 4.3. Exploring resistance to initial chemotherapy in SCLC

#### 4.3.1. Molecular subtypes of SCLC

The mechanistic analysis of drug resistance has revealed that SCLC’s high heterogeneity and pronounced subtype plasticity are the primary contributors to resistance against chemotherapy and immunotherapy.^[19,28–31]^ Different SCLC subtypes exhibit varied therapeutic vulnerabilities, with the inflamed subtype showing the most promising response to immunotherapy. This underscores the importance of precision medicine in clinical settings.^[19]^

Recent advances in SCLC research have identified 4 major molecular subtypes based on the differential expression of lineage-related transcription factors: SCLC-A (characterized by high expression of achaete-scute homolog 1, ASCL1), SCLC-N (high neurogenic differentiation factor 1, NEUROD1), SCLC-Y (high YES-associated protein 1, YAP-1), and SCLC-P (high POU class 2 homeobox 3, POU2F3).^[32–34]^ ASCL1 directly influences the expression of delta-like ligand 3 (DLL3), which is found predominantly in SCLC-A and minimally expressed in normal tissues,^[35]^ providing a target for SCLC-specific therapeutics.^[31]^ The DLL3-targeting antibody–drug conjugate, rovalpituzumab tesirine, has undergone clinical trials,^[36]^ although its phase II study indicated significant associated toxicities.^[37]^ However, new DLL3-targeting approaches are currently in development.^[38]^

Up-regulation of C-MYC, associated with the SCLC-N subtype,^[33]^ represents a potential target for therapeutic intervention.^[39,40]^ Sensitivity to aurora kinase inhibitors is unique in SCLC driven by C-MYC, with a recent double-blind clinical study confirming C-MYC as a potential predictive biomarker for response to the aurora A inhibitor alisertib.^[41,42]^ The SCLC-Y subtype, noted for its inflamed tumor microenvironment, may particularly benefit from immune checkpoint blockade therapy.^[43]^ In contrast, the SCLC-P subtype has shown potential responsiveness to targeted therapy with the myeloid cell leukemia 1 inhibitor.^[44]^

The overall efficacy of immunotherapy in unselected SCLC patients remains modest, highlighting the urgent need to discern which patients might benefit the most from such treatments. Recently identified subtypes, SCLC-Ia and SCLC-Ib, further refine this stratification. SCLC-Ia, characterized by low expression of ASCL1, NEUROD1, and POU2F3 along with an inflammatory gene signature, is sensitive to the addition of immunotherapy to chemotherapy. SCLC-Ib, marked by immunosuppressive characteristics and high genomic instability, shows that patients with high POU2F3 expression respond better to immunotherapy.^[28,45]^ These findings provide a clinically relevant method to distinguish SCLC patients more likely to benefit from immunotherapies.

Different SCLC subtypes exhibit varying susceptibilities to targeted therapies, including inhibitors of the nuclear enzyme poly (ADP-ribose) polymerase (PARPi).^[46]^ The clinical benefits of PARPi monotherapy are restricted to a specific subset of SCLC patients, such as those with high expression of SLFN11.^[47]^ When combined with DNA-damaging agents (e.g., cisplatin and etoposide, temozolomide, camptothecin, irinotecan), PARPi has demonstrated promising efficacy and a tolerable safety profile.^[48–50]^ However, such combined therapies, designed to maximize DNA damage, have been associated with increased toxicity.^[51]^ The clinical response to the combination of chemotherapy and PARP inhibitors has been modest in patients with relapsed small-cell carcinoma. It is crucial to recognize that patients who exhibit specific biomarkers (e.g., high E2F1 expression,^[47]^ elevated MGMT methylation,^[48]^ high SLFN11 expression,^[49]^ and platinum sensitivity^[50]^) show better treatment responses, underscoring the importance of predictive biomarkers in tailoring treatment regimens.

#### 4.3.2. Bone metastasis and chemosensitivity

Our study found, through binary logistic regression analysis, that bone metastasis was an independent high-risk factor for chemosensitivity. Bone metastases often exhibit resistance to chemotherapy due to the bone marrow–blood barrier. Although various drugs have been developed for bone-related disorders, delivery to the bone marrow remains a challenge. Luo et al^[52]^ reported using neutrophils for the targeted delivery of free drugs and drug nanoparticles to the bone marrow. In a model of bone metastasis, neutrophil-mediated delivery of cabazitaxel significantly inhibited tumor growth, suggesting potential applicability in treating various bone diseases.

##### 4.3.2.1. Osteoimmuno-oncology

The emerging field of osteoimmuno-oncology focuses on the interaction between cancer cells, bone, and immune cells.^[53]^ A thorough understanding of these interactions is crucial to developing effective and safe treatments for bone metastases in cancer patients.^[54]^ A recent study indicated that musculoskeletal adverse effects, commonly observed in patients receiving immunotherapy, were positively correlated with antitumor responses.^[55]^ This observation suggests that immunotherapy could offer new hope for patients with bone metastases.^[56]^ However, there are few reports of approved immunotherapies in breast, prostate, and lung cancers that specifically address outcomes in bone metastases. For example, patients with advanced or metastatic triple-negative breast cancer treated with atezolizumab and nab-paclitaxel exhibited a longer median overall survival compared to those who received placebo and nab-paclitaxel.^[57]^ Given the low response rates and the shorter progression-free and overall survival rates associated with bone metastases,^[58]^ it is imperative that clinical trials specifically evaluate the impact of immunotherapies on bone metastases and consider bone metastasis as a crucial stratification factor.

##### 4.3.2.2. PT-112

Imifoplatin (PT-112), a cytotoxic platinum–pyrophosphate compound, has been studied for its efficacy in cancer treatment, particularly in the context of the bone metastatic microenvironment.^[54]^ Due to its pyrophosphate component, PT-112 is highly concentrated in bone tissues.^[59]^ In a combination study (NCT03409458) with avelumab targeting metastatic castration-resistant prostate cancer, significant therapeutic activity was observed: 24 of 32 patients exhibited lower serum alkaline phosphatase levels along with improvements in pain and quality of life.^[54,60]^

PT-112 has demonstrated dose-proportional pharmacokinetics and was well tolerated. It showed promising drug activity in various cancers including small cell and non-small cell lung cancer, as well as in prostate cancer among a nonselected, heavily pretreated cohort. This includes cases with long-duration stabilization and response of the disease, even among patients who did not respond to prior immune checkpoint inhibitors.^[61]^ This suggests that PT-112 may induce an adaptive immune response consistent with immunogenic cancer cell death. The drug is associated with an increase in relevant immune cell populations, such as dendritic cells and cytotoxic/helper T cells, in preclinical models, which contributes to the recruitment of tumor-infiltrating lymphocytes.^[54,61]^ Its combination with anti-PD-L1 therapy in metastatic castration-resistant prostate cancer patients underscores the rationale for such therapeutic partnerships, showcasing a promising outlook for the development and application of immuno-oncology drugs focused on bone metastasis.^[60]^

#### 4.3.3. Limitations

This study has several limitations. First, it is a single-center retrospective analysis with a small sample size, which may introduce confounders and limit the generalizability of the findings. Second, all the cases included in this study are from the Chinese population, which has certain limitations in terms of racial demographic information. Third, the subset of SCLC patients with insensitivity to initial chemotherapy was relatively small, which could affect the robustness of the subgroup analyses. Fourth, immunohistochemical profiling was incomplete, particularly with missing data on key neuroendocrine markers. This absence could impede a comprehensive understanding of their association with clinicopathologic chacteristics and chemosensitivity.

## 5. Conclusions

Bone metastasis was identified as an independent high-risk factor for insensitivity to initial platinum-based first-line chemotherapy in patients with SCLC. Advancing our understanding of SCLC biology and osteoimmuno-oncology is crucial to identifying novel vulnerabilities that could be targeted in clinical treatments to overcome drug resistance. Gene expression profiling has defined distinct molecular subtypes of SCLC, demonstrating unique susceptibilities to targeted or immune therapies. Integration of molecularly targeted therapy or immunotherapy with traditional chemotherapy holds promise in improving clinical outcomes. Tailoring treatment strategies to the specific molecular characteristics of SCLC can significantly enhance patient responses and survival rates.

## Author contributions

**Conceptualization:** Yulong He, Wenwang Lang.

**Data curation:** Yulong He, Xiaorong Tang, Fang Yang, Qinling Jiang, Lin Deng.

**Formal analysis:** Yulong He.

**Methodology:** Yulong He, Qinling Jiang.

**Resources:** Yulong He, Lin Deng.

**Software:** Yulong He, Xiaorong Tang, Qinling Jiang, Lin Deng, Wenwang Lang.

**Writing – original draft:** Yulong He.
